# YTHDF1 promotes gallbladder cancer progression via post‐transcriptional regulation of the m6A/UHRF1 axis

**DOI:** 10.1111/jcmm.18328

**Published:** 2024-04-29

**Authors:** Jiemin Chen, Xuesong Bai, Wenqin Zhang, Zhiyu Yan, Yongru Liu, Shengnan Zhou, Xi Wu, Xiaodong He, Aiming Yang

**Affiliations:** ^1^ State Key Laboratory of Complex Severe and Rare Diseases, Department of Gastroenterology Peking Union Medical College Hospital, Peking Union Medical College and Chinese Academy of Medical Sciences Beijing China; ^2^ State Key Laboratory of Complex Severe and Rare Diseases, Department of General Surgery Peking Union Medical College Hospital, Peking Union Medical College and Chinese Academy of Medical Sciences Beijing China

**Keywords:** cancer progression, gallbladder cancer, m6A modification, UHRF1, YTHDF1

## Abstract

Gallbladder cancer is a rare but fatal malignancy. However, the mechanisms underlying gallbladder carcinogenesis and its progression are poorly understood. The function of m6A modification and its regulators was still unclear for gallbladder cancer. The current study seeks to investigate the function of YTH m6A RNA‐binding protein 1 (YTHDF1) in gallbladder cancer. Transcriptomic analysis and immunochemical staining of YTHDF1 in gallbladder cancer tissues revealed its upregulation compared to paracancerous tissues. Moreover, YTHDF1 promotes the proliferation assays, Transwell migration assays, and Transwell invasion assays of gallbladder cancer cells in vitro. And it also increased tumour growth in xenograft mouse model and metastases in tail vein injection model in vivo. In vitro, UHRF1 knockdown partly reversed the effects of YTHDF1 overexpression. Mechanistically, dual‐luciferase assays proved that YTHDF1 promotes *UHRF1* expression via direct binding to the mRNA 3′‐UTR in a m6A‐dependent manner. Overexpression of YTHDF1 enhanced UHRF1 mRNA stability, as demonstrated by mRNA stability assays, and Co‐IP studies confirmed a direct interaction between YTHDF1 and PABPC1. Collectively, these findings provide new insights into the progression of gallbladder cancer as well as a novel post‐transcriptional mechanism of YTHDF1 via stabilizing target mRNA.

## 1 INTRODUCTION

Gallbladder cancer (GBC) is a rare malignancy with high incidence in East Asia. In 2022, approximately 30,000 new cases and 25,000 deaths were reported in China,^1^ while approximately 4800 new cases and 2400 deaths were reported in the United States.^1^, ^2^ GBC diagnosis is often an incidental finding that occurs during cholecystectomies, with metastases often occurring early,^3^, ^4^ resulting in a relatively poor prognosis for patients with metastatic GBC.^5^ Meanwhile, curative treatment often requires radical dissection.^4^, ^5^ However, the mechanism underlying the development and progression of GBC remains poorly understood.

Recently, defining the role of m6A modifications in cancer research has become an area of interest. The YTH domain family proteins play a significant role as m6A readers, recognizing m6A modifications and acting as post‐transcriptional regulators. YTHDF1 acts as an m6A reader that directly initiates cap‐independent translation,^6^ and functions as an oncogene or tumour suppressor in various malignancies, including hepatocellular carcinoma,^7^ breast cancer^8^ and colorectal cancer.^9^ However, the biological functions and mechanisms of YTHDF1 and m6A modifications in GBC remain unclear.

Dysregulation of m6A or the aberration of its modulator proteins also contributes to cancer formation.^10^ Crosstalk between RNA methylation, super‐enhancer RNA and DNA m5C modifications has been reported previously.^11^, ^12^ UHRF1 regulates various epigenetic markers, including repressive epigenetic markers, such as m5C methylation, as well as active chromatin mark, such as H3K4me3, leading to transcription factor‐like activity.^13^ Previous study revealed that depletion of UHRF1 promoted apoptosis,^14^, ^15^ cell cycle arrest,^14^ metastases,^16^ epithelial‐mesenchymal transition,^17^ and acted as an oncogene in multiple cancers, including small cell lung cancer,^18^ pancreatic cancer,^19^ intrahepatic cholangiocarcinoma^20^ and GBC.^14^

In this study, we suppose to investigate the clear functions of YTHDF1 and m6A‐dependent functions on regulating m6A/UHRF1 axis in gallbladder cancer progression. High expression of YTHDF1 stabilized UHRF1 mRNA via an m6A‐dependent manner by recruiting PABPC1. Our finding suggested a novel mechanism of YTHDF1/PABPC1 as a stabilizer of the UHRF1 mRNA and promoting GBC progression.

## 2 MATERIALS AND METHODS

### 2.1 Patient samples

FFPE GBC samples were collected from 135 patients diagnosed with gallbladder cancer (GBC) who underwent cholecystectomy between 2015 and 2020 at Peking Union Medical College Hospital. Patients included in the study had not received any prior radiotherapy or chemotherapy. All patients provided informed consent before undergoing surgery. The baseline data of the enrolled patients were provided in the Table S1. We also collected 6 pairs of frozen tissue specimens from GBC and corresponding adjacent tissues for RNA sequencing.

### 2.2 Plasmid construction, lentiviral packaging and concentration

The coding sequence of YTHDF1 mRNA and UHRF1 mRNA was PCR amplified, digested with EcoRI and BamHI, and ligated to vector pLVX‐CMV‐EGFP‐T2A‐PuroR as pLVX‐YTHDF1 and pLVX‐UHRF1. shYTHDF1 oligonucleotides were annealed and ligated into the vector pSIH‐H1‐copGFP‐T2A‐Puro as pSIH‐shYTHDF1‐1/‐2. The ligated products were transformed into competent *Escherichia coli* DH5α cells (Vazyme, Nanjing, China), cultured, and extracted with the QIAGEN EndoFree™ Plasmid Extraction Kit (12362, QIAGEN, Germany). pLVX‐YTHDF1, pLVX‐UHRF1, pSIH‐shYTHDF1‐1/2 together with psPAX2, pMD2.G was transfected by polyethyleneimine linear (PEI) (MW = 40,000, Sigma), respectively, as described previously.^21^ After 48 h, supernatants from the cultures were collected and mixed with 0.4 M NaCl and 12% PEG‐6000 overnight, followed by centrifugation at 6000×*g* for 15 min. Virus pellets were resuspended by 200 μL 1× PBS for further use. The oligonucleotides used in this study was listed in Table S2.

### 2.3 Construction of stably‐expressing cell lines

The GBC‐SD (RRID: CVCL_6903) and HEK‐293FT (RRID: CVCL_6911) cell lines were obtained from the Cell Resource Center, Peking Union Medical College. The NOZ (RRID: CVCL_3079) cell line was obtained from the JCRB Cell Bank (Tokyo, Japan). GBC‐SD cells were cultured in RPMI‐1640 medium supplemented with 10% fetal bovine serum (FBS; Gibco) and 1% penicillin–streptomycin (Gibco). HEK‐293FT cells were cultured in DMEM‐H supplemented with 10% FBS (10099‐141C, Gibco, USA) and 1% penicillin–streptomycin (Gibco, USA). NOZ cells were cultured in William's E medium supplemented with 10% FBS (10099‐141C, Gibco) and 1% penicillin–streptomycin (Gibco).

All cell lines have been authenticated by STR profiling. Briefly, the Microreader™21 ID System (Microreader, China) was utilized for PCR, then ABI 3130XL DNA Analyser (ThermoFisher, USA) and GeneMapper3.2 software was used to compare the results with the Cellosaurus database for reference matching. All cell lines were verified to be mycoplasma‐free using the MycoBlue Mycoplasma Detector kit (Vazyme).

The GBC‐SD and NOZ cell lines were passaged and seeded in 96‐well plates at 70%–90% confluence. The lentiviruses were added to the culture medium at an average multiplicity of infection (MOI) of five. After 24 h, 6 μg/μL puromycin was added to the medium and passaged an additional 2–3 times.

### 2.4 RNA extraction, reverse transcription and real‐time PCR

Total RNA was extracted using the Eastep Super Total RNA Extraction Kit (LS1030; Promega, USA) according to the manufacturer's instructions. DNase I was included in the kit. Reverse transcription was performed by GoScript™ Reverse Transcription Mix, oligo‐dT (A2798, Promega, USA) at 42°C for 60 min, followed by heat inactivation at 80°C for 10 min. Real‐time PCR was performed by GoTaq SYBR Master Mix (A6001, Promega, USA) using the QuantStudio™ 5 Real‐time PCR System (ThermoFisher, USA). Relative quantification was calculated by the 2^−ddCt^ method, with ACTB mRNA as the standard. Primers used for real‐time PCR are listed in Table S3.

### 2.5 Protein extraction and Western blot

Cultured cells were washed twice with 1 × PBS and then harvested by adding 100 μL NP‐40 lysis buffer with 1× proteinase inhibitor cocktail (MCE, USA). Protein samples were electrophoresed with 10% NuPAGE Bis‐Tris mini gels at 200 V in 1× MOPS running buffer. The gels were then semi‐dry transferred onto 0.22 μm nitrocellulose (NC) membranes using a Trans‐Blot™ Turbo semi‐dry transfer machine (Bio‐Rad, USA). The transferred NC membranes were blocked in 5% skimmed milk for 1 h, then primary antibodies were added and incubate overnight at 4°C with gentle shaking. For chemiluminescent detection, HRP‐conjugated anti‐rabbit secondary antibody was diluted 1:10,000, added and incubated for 1 h at room temperature. ChemiDoc MP (Bio‐Rad, USA) was used for chemiluminescent signal detection, using SuperSignal West Pico chemiluminescent substrate (#34080, ThermoFisher, USA). 16‐bit TIFF images were acquired and analysed using Bio‐Rad Image Lab Software ver. 6.1 (Bio‐Rad Inc., USA). The antibodies used in this study are listed in Table S4.

### 2.6 Transcriptomic analyses

Total RNAs of paired GBC tissues were extracted using the Eastep Super Total RNA Extraction Kit (LS1030; Promega, USA) according to the manufacturer's instructions. rRNA was depleted using the NEBNext® rRNA Depletion Kit (E6310, NEB, USA). Fragmentation, first‐strand synthesis, second‐strand synthesis, end repair, USER digestion and PCR amplification were performed using the NEBNext Ultra II Directional RNA Library Prep Kit for Illumina (E7760, NEB, USA). The dsDNA library was quantified using a Qubit 4 fluorometer and qualified using an Agilent 2100 analyser. NGS sequencing was performed by Beijing Novogene Bioinformatics Technology Co. Ltd., using a NovaSeq™ platform. Adaptor sequences were trimmed, and raw data were filtered using fastp (v0.21.0, https://github.com/OpenGene/fastp). Clean data were mapped to the reference genome GRCh38 and v108 annotations using the RNA STAR aligner (2.7.4a, https://github.com/alexdobin/STAR/).^22^ The featureCounts (v2.0.3, https://subread.sourceforge.net/featureCounts.html) program was used to quantify gene expression.^23^ The fold changes and *p*‐values of differentially expressed genes (DEGs) were calculated using DESeq2.^24^ Gene ontology (GO) enrichment analyses and Kyoto Encyclopedia of Genes and Genomes (KEGG) enrichment analyses were performed using R package clusterProfiler.

### 2.7 m6A/RNA‐immunoprecipitation and sequencing (m6A‐seq/RIP‐seq)

Total RNA was extracted using the Eastep Super Total RNA Extraction Kit (Promega, USA) according to the manufacturer's instructions. Total RNA was fragmented using the NEBNext Magnesium RNA Fragmentation Module (#E6150, New England Biolabs) at 94°C for 10 min. Next, either 5 μg of anti‐m6A antibody (1:50, ab151230, Abcam, USA) or anti‐YTHDF1 antibody (17479‐1‐AP, Proteintech, Wuhan, China) and IgG isotype control antibody (#3900S, Cell Signal Technology, USA) were conjugated to 100 μL of pre‐cleaned Protein G Dynabeads for overnight mixing at 4°C. Antibody‐conjugated beads were mixed with total RNA in NT2 Wash Buffer (10 mM Tris–HCl, 150 mM NaCl, 2.5 mM MgCl_2_, 0.1% Igepal CA‐630) on a rotator at 4°C for 2 h. The beads were then sequentially washed with NT2 wash buffers. Proteinase K was added, and the cells were incubated at 37°C for 30 min. The enriched RNA was further purified from the lysate using an RNeasy MinElute Cleanup Kit (QIAGEN, Germany). The NEBNext Ultra II Directional RNA Library Prep Kit from Illumina (New England Biolabs, Ipswich, MA, USA) was used to prepare the RNA library. For m6A‐seq, peak analysis was performed using the R package exomePeak,^25^ and read coverage was analysed and illustrated using bedtools and the R package Gviz. For RIP‐seq, data were processed in the same pipeline as described in transcriptome analysis. Enrichment scores were calculated as fold change in IP/10% input and *p*‐values were calculated using Student's *t*‐test.

### 2.8 Cell proliferation assay

pLVX‐Control, pLVX‐YTHDF1, pSIH‐Control, pSIH‐shYTHDF1‐1 and pSIH‐shYTHDF1‐2 stably expressing NOZ and GBC‐SD cells were seeded at 1000 cells/well in 96‐well plates. Using a Synergy H1 microreader connected to a Biospa8 automated incubator (Agilent, USA), a series of photographs were automatically captured of each well, and vial cell counts were calculated every 24 h for 5 days. Growth curves are shown in the figures as the cell counts in each well at 0, 24, 48, 72 and 96 h. Two‐way ANOVA was used to compare groups, and Tukey's multiple comparisons test was used to evaluate differences between groups.

### 2.9 Cell apoptosis assay

pLVX‐Control, pLVX‐YTHDF1, pSIH‐shControl, pSIH‐shYTHDF1‐1 and pSIH‐shYTHDF1‐2 stably expressing NOZ and GBC‐SD cells were seeded in 6‐well plates. Cells were treated with 10 μM CCCP and incubated at 37°C and 5% CO_2_ for 16 h. The cells were then dissociated from the plates using 0.25% trypsin–EDTA solution. Cells in suspension were stained using the Annexin V‐Alexa Fluor™ 633/7‐AAD staining kit (AD11, Dojindo, Japan) according to the manufacturer's instruction. The apoptosis assay was performed using an Attune NXT cytometer (Thermo Fisher Scientific) with BL2/RL3 channels. Raw FCS data were exported and analysed using FlowJo v10.4 (BD, USA). Changes in the apoptosis rates were compared using the Chi‐square tests with Bonferroni correction.

### 2.10 Wound healing assay

NOZ and GBC‐SD cell lines transfected with pLVX‐Control, pLVX‐YTHDF1, pSIH‐Control, pSIH‐shYTHDF1‐1, or pSIH‐shYTHDF1‐2 were seeded in 12‐well plates and subjected to wound‐healing assays (#80209, Ibidi, Germany). Briefly, 500 μm wounds were generated by removing the cell assay inserts from the plates. Cells were treated with serum‐free culture medium and incubated continuously for 48 h at 37°C and 5% CO_2_ in a BioSpa8 Automated Incubator (BioTek, USA). Images of the wound sites were captured every 24 h using a Cytation 7 cell‐imaging multimode reader (BioTek). The ratio of the areas was measured using ImageJ software, and the differences were calculated using the chi‐square tests with Bonferroni correction.

### 2.11 Transwell migration assay and invasion assay

NOZ and GBC‐SD cell lines stably expressing pLVX‐Control, pLVX‐YTHDF1, pSIH‐Control, pSIH‐shYTHDF1‐1 and pSIH‐shYTHDF1‐2 were suspended in serum‐free culture medium at a concentration of 5 × 10^5^/mL. Next, 600 μL of culture medium containing 20% FBS was added to a 24‐well plate. Uncoated Transwell inserts for the migration assay and 0.5% Matrigel (356234, Corning)‐coated Transwell inserts for the invasion assay were placed in each well. A 400 μL suspension of the cell line was seeded and incubated for 24 h. The upper side of the cell layer was then swabbed, and the lower side of the Transwell inserts was incubated in 4% paraformaldehyde for 30 min and stained with 0.5% crystal violet overnight. Photographs of the Transwell inserts were captured using a Leica DM IL (Leica, Germany) inverted microscope and Leica K3C digital camera (Leica, Germany). Cells were counted and compared between the groups.

### 2.12 Animal model

Twenty‐five 6‐week‐old male BALB/c nude mice were purchased from Beijing Vitalstar Biotechnology Co., Ltd. (Beijing, China) and housed at the Institute's Animal Faculty. They were randomly divided into five groups: shControl, shYTHDF1‐1, and shYTHDF1‐2; NC, YTHDF1. A suspension containing 5 × 10^6^ cells was injected subcutaneously into each nude mouse and tumour volumes were measured every other day. After 28 days, or before tumour volume exceeds 2000 mm^3^, the animals were sacrificed by CO_2_ inhalation, and subcutaneous tumours were harvested.

Twenty 6‐week‐old male NOD/SCID mice were randomly divided into four groups: NC, shYTHDF1‐1, shYTHDF1‐2 and YTHDF1. A suspension containing 5 × 10^5^ cells was intravenously injected into the tail vein of each mouse. After 28 days, the animals were sacrificed. The lungs were harvested, and the number of tumours was calculated. All animals were housed in an SPF environment with free access to food and 12‐h light–dark cycles.

### 2.13 Immunohistochemical staining

Briefly, 4‐μm‐thick sections of formalin‐fixed, paraffin‐embedded (FFPE) tissue blocks were heated in a 60°C oven for 1 h. Sections were deparaffinized and rehydrated prior to staining. Antibody retrieval was performed using pH 7 or pH 9 antibody retrieval solution (Solarbio, Beijing, China) using a microwave oven. Sections were then blocked with 10% goat serum for 2 h at room temperature. Primary antibodies were then incubated overnight at 4°C. The sections were then rinsed with gentle agitation for 10 min in TBS‐0.025% Triton X‐100. HRP‐conjugated secondary antibodies were added to the sections and incubate for 1 h at room temperature. Finally, sections were incubated with 1× DAB for 10 min at room temperature and rinsed under running water for at least 3 min. The antibodies and concentration used in this study are listed in Table S4.

### 2.14 Dual‐luciferase assay

Sequences with wild‐type or mutant binding sites were cloned into the 3′‐UTR region of firefly luciferase in the pmirGLO vector (Promega, USA) using restriction enzymes XbaI and XhoI and T4 ligase. The primer and oligonucleotide sequences are listed in Table S1. Ligated products were transformed into super‐competent *E*. *coli* DH5α cells (Vazyme, Nanjing, China), cultured, and extracted with the QIAGEN EndoFree™ Plasmid Extraction Kit (12362, QIAGEN, Germany). The vectors carrying Seq 1‐WT, Seq 1‐Mut were transfected into Vector and YTHDF1‐overexpressing cell lines using the Lipofectamine 3000 transfection reagent (Thermo Fisher Scientific, USA), according to the manufacturer's instructions. After 72 h of incubation, the luciferase activity was measured using a Dual‐Glo Luciferase Assay System (Promega, USA) and a Synergy H1 microplate reader (Thermo Fisher, USA). The normalized ratio of firefly luciferase to Renilla luciferase was calculated to assess the regulation of the target genes.

### 2.15 Co‐immunoprecipitation (Co‐IP)

Either 5 μg of anti‐PABPC1 antibody (10970‐1‐AP, Proteintech, Wuhan, China), anti‐YTHDF1 antibody (17479‐1‐AP, Proteintech, Wuhan, China) and IgG isotype control antibody (#3900S, Cell Signal Technology, USA) were conjugated to 100 μL of pre‐cleaned Protein G Dynabeads for overnight mixing at 4°C. Total protein of 10‐cm‐dish cultured cells was extracted using 400 μL IP Lysis buffer (10 mM Tris–HCl, 150 mM NaCl, 0.5% Igepal CA‐630). 10% Input samples were collected for further use. Antibody‐conjugated beads washed with twice 600 μL NT2 Wash Buffer (10 mM Tris–HCl, 150 mM NaCl, 2.5 mM MgCl_2_, 0.1% Igepal CA‐630), then mixed with total protein on a rotator at 4°C for 2 h. The beads were then sequentially washed with twice 600 μL NT2 wash buffers. 4× NuPAGE™ LDS Sample Buffer (NP0007, ThermoFisher, USA) and 10× NuPAGE™ Sample Reducing Agent (NP0009, ThermoFisher, USA) was added to 200 μL PBS dissolved beads, and the cells were heated at 70°C for 15 min. Then beads were then placed on magnetic stands and supernatant protein solution was moved to new EP tubes. Western blotting of 10% Input, IgG and IP was performed and expression levels were calculated using Bio‐Rad ImageLab version 6.1.

### 2.16 Actinomycin D‐dependent mRNA stability assay

Cells were plated in five 6‐well plate at confluence between 70%–90%. Actinomycin D (5 μg/mL) was added in culture medium. Cells were harvested at 0, 2, 4, 6 and 8 h. Total RNA of cell pellets was isolated using the Eastep Super Total RNA Extraction Kit (LS1030, Promega, USA) according to the manufacturer's instructions. Reverse transcription was carried out using 2 μg of total RNA via GoScript™ Reverse Transcription Mix, oligo‐dT (A2798, Promega, USA) at 42°C for 60 min, followed by heat inactivation at 75°C for 15 min. The QuantStudio™ 5 Real‐time PCR System (ThermoFisher, USA) was used to run real‐time PCR with GoTaq SYBR Master Mix (A6001, Promega, USA). Relative quantification was calculated by the 2^−ddCt^ method, which was standardized to ACTB mRNA. Log2‐transformed expression level of UHRF1 mRNA and incubation time was fitting to an exponential growth curve using GraphPad version 8.0. For each group, the half‐time period and R‐square were calculated and illustrated.

## 3 RESULTS

### 3.1 YTHDF1 serves as a prognostic factor and is upregulated in gallbladder cancer tissues

**FIGURE 1:**
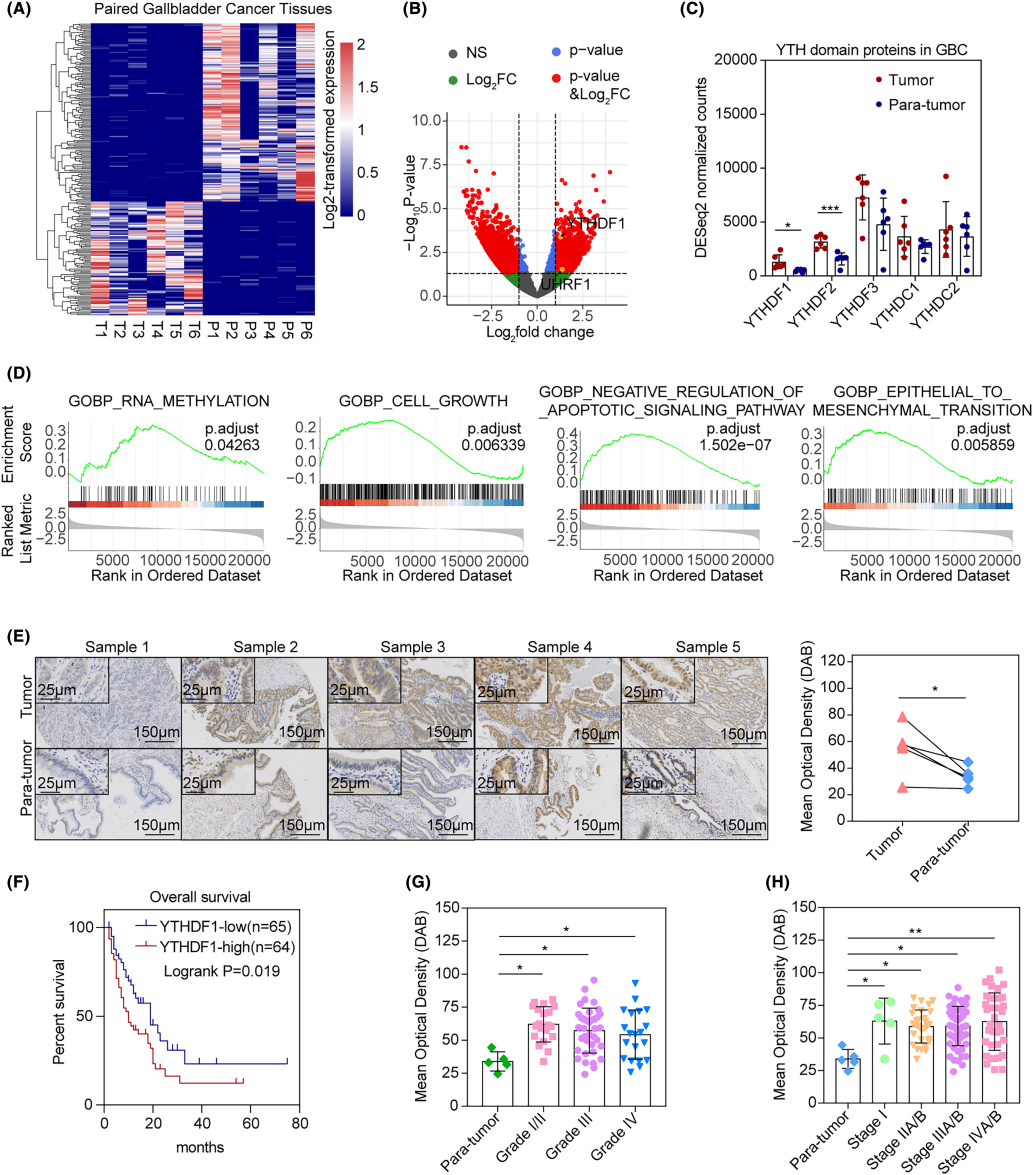
(A) Transcriptomic analysis of six paired GBC tissues. DESeq2 normalized expression value was log2‐transformed and significant genes with adjusted *p* < 0.05 were illustrated; (B) volcano plot demonstrating DEGs in RNA‐seq of six paired GBC tissues; (C) DESeq2 normalized expression value of YTH domain family proteins in paired GBC tissues. *p < 0.05, ***p < 0.001; (D) GSEA enrichment analyses of six paired GBC tissues' RNA‐seq. Selected enriched pathways and an adjusted *p*‐value were reported; (E) (left) immunohistochemical staining of YTHDF1 in five paired gallbladder cancer (GBC) tissues. The 50× and 200× images are illustrated; (right) mean optical densities (MOD) of IHC staining of YTHDF1 in five paired GBC tissues were measured. A paired *t*‐test was used to compare the two groups. *p < 0.05; (F) Kaplan–Meier survival analysis of 129 patients divided by median value of YTHDF1 MODs. The logrank test was performed to compared difference of overall survival between two groups; (G) MODs of IHC staining for YTHDF1 in tissue microarray between pathological grading of 135 GBC samples. ANOVA test and Tukey's multiple comparison tests were used to compare between groups. **p < 0.01; (H) MODs of IHC staining for YTHDF1 in 135 GBC samples between clinical stages in tissue microarray. ANOVA test and Tukey's multiple comparison tests were used to compare between groups. **p < 0.01.

Transcriptional analyses were conducted on six pairs of GBC tissues, identifying 236 genes with significant differences (Figure 1A,B). In cancer tissues, both YTHDF1 (*p* < 0.05) and YTHDF2 (*p* < 0.001) show significant upregulation compared to para‐cancerous tissues (Figure 1C), with YTHDF1 exhibiting a more pronounced increase in fold changes (Log_2_FC = 1.31 vs. 0.98).

Cell growth (*p* < 0.05), negative regulation of the apoptotic signalling pathway (*p* < 0.0001), epithelial‐mesenchymal transition (*p* < 0.01), and RNA methylation (*p* < 0.05) were each significantly enriched in GSEA enrichment analyses (Figure 1D).

FFPE sections with gallbladder cancer (GBC) were evaluated to determine the mean optical density (MOD) of anti‐YTHDF1 immunohistochemical staining. In five pairs of matched tumour and para‐tumour tissues, the MOD of the cancerous tissue was significantly upregulated (*p* < 0.05, Figure 1E).

A total 129 GBC patients with available clinical data were divided into two groups according to the YTHDF1 MOD: YTHDF1‐high (*n* = 64) and YTHDF‐low (*n* = 65). The baseline data of two groups were provided in Table S1. Kaplan–Meier analyses and logrank test proved that YTHDF1‐high expression group had inferior overall survival (*p* < 0.05, Figure 1F).

In non‐paired cancerous tissues from patients with GBC, the MOD of pathologic grades and differed with that of Grade I/II (*p* < 0.05), Grade III and Grade IV (*p* < 0.05) found to be significantly higher than that of para‐tumour tissues (Figure 1G). The MOD of clinical stages differed with that of Stage I (*p* < 0.05), Stage IIA/B (*p* < 0.05), Stage IIIA/B (*p* < 0.05) and Stage IVA/B (*p* < 0.01) found to be significantly higher than that of para‐tumour tissues (Figure 1H).

### 3.2 YTHDF1 promotes malignant behaviour in gallbladder cancer cell‐lines

To investigate whether *YTHDF1* altered the malignant behaviours of GBC cells, we employed a *YTHDF1* overexpression vector and two distinct short hairpin RNA vectors (shYTHDF1‐1 and shYTHDF1‐2) to alter *YTHDF1* expression in NOZ and GBC‐SD cell lines.

**FIGURE 2:**
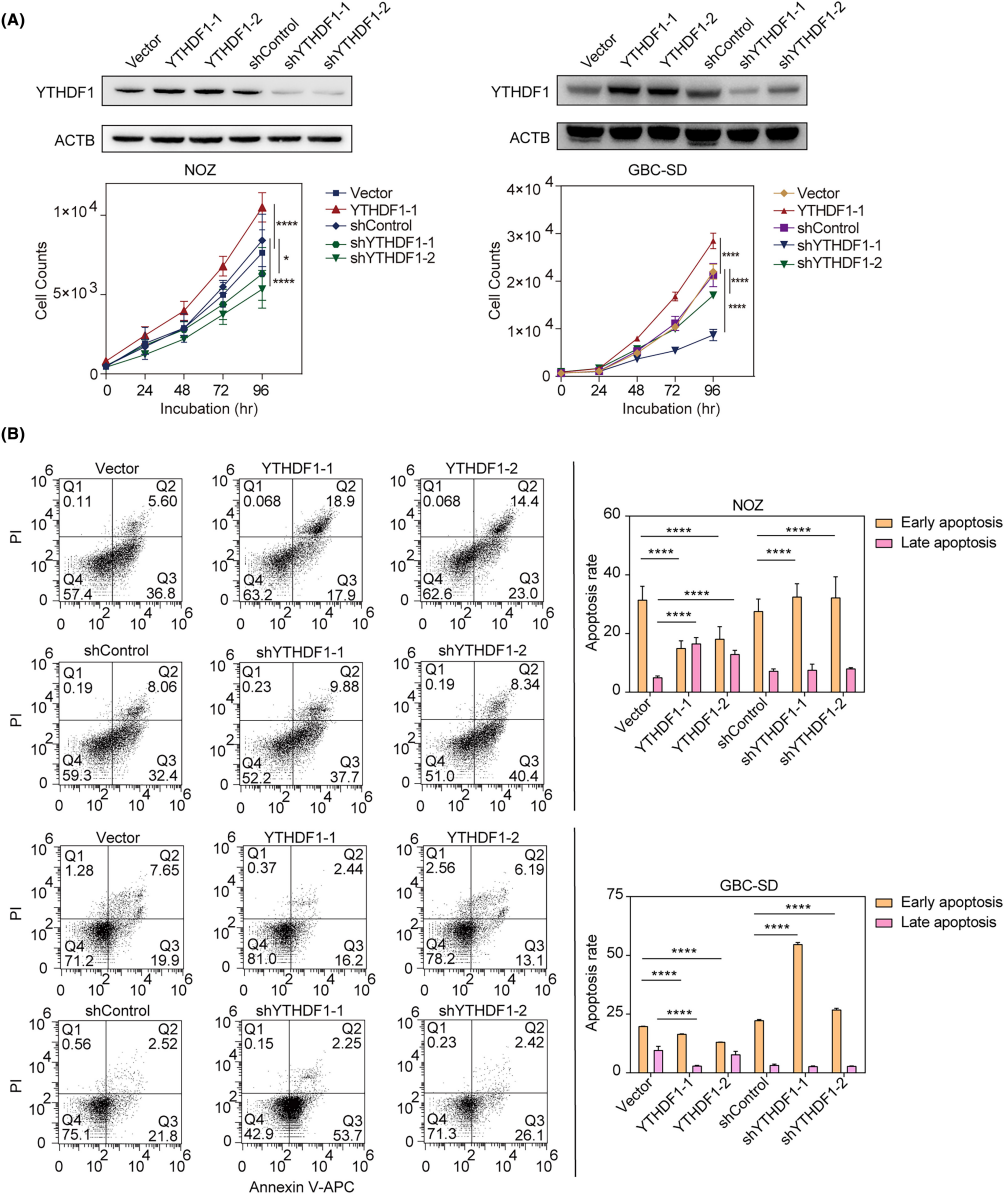
(A) (up) YTHDF1 overexpressed and knocked‐down cell lines used in phenotype experiments were validated using Western blotting; YTHDF1‐1 and YTHDF1‐2 were technical repeats; shYTHDF1‐1 and shYTHDF1‐2 were biological repeats; (down) proliferation assay for NOZ and GBC‐SD cell lines. Cell counts were automatically recorded, analysed and illustrated. Two‐way ANOVA was used to compare multiple curves and Tukey's multiple comparison tests were used to compare groups. The illustrated results were representative of three replicates. *p < 0.05, ****p < 0.0001; (B) apoptosis assay for NOZ and GBC‐SD cell lines. Early apoptosis (Q3) and late apoptosis (Q2) was compared between groups using Chi‐square tests with Bonferroni correction. The illustrated results were representative of three replicates. ****p < 0.0001.

*YTHDF1* overexpression significantly increased cancer cell proliferation when compared to Vector control group (*p* < 0.0001), while its knockdown decreased the proliferation of NOZ and GBC‐SD cells (*p* < 0.0001, Figure 2A).

Overexpression of *YTHDF1* (YTHDF1‐1 and YTHDF1‐2) also decreased the CCCP‐induced early apoptosis rate when compared with Vector group, while its knockdown (shYTHDF1‐1 and shYTHDF1‐2) promoted CCCP‐induced early apoptosis when compared with shControl group in both NOZ and GBC‐SD cell lines (Figure 2B).

**FIGURE 3:**
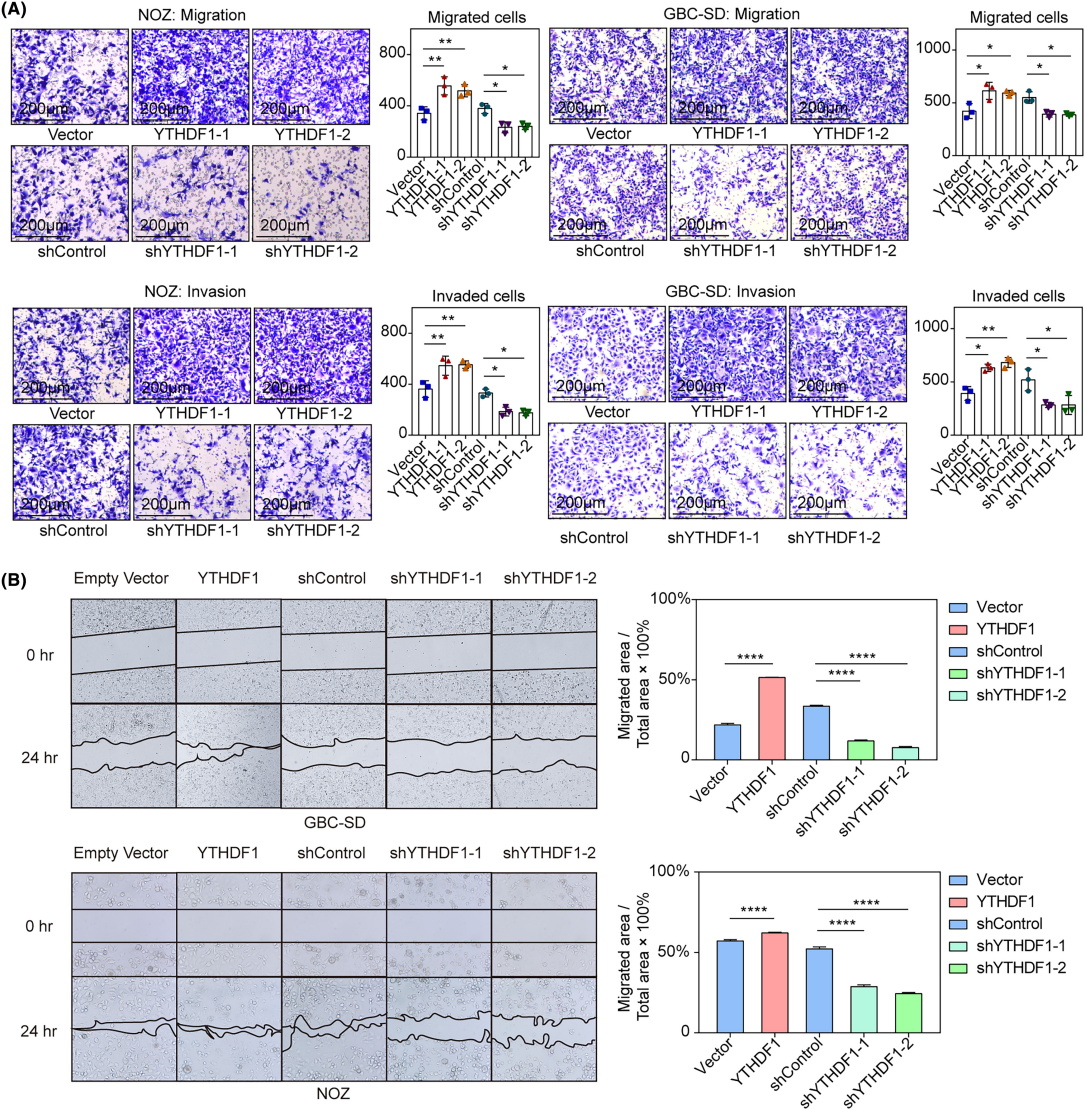
(A) Transwell migration and invasion assays for NOZ and GBC‐SD cell lines. Migrated cells were counted and compared between groups. Comparisons between groups were made using ANOVA test and Tukey's multiple comparison tests. The illustrated results were representative of three replicates. *p < 0.05, **p < 0.01, ***p < 0.001; (B) wound healing assay for NOZ and GBC‐SD cell lines. Migrated area/total area were measured and compared between groups using the Chi‐square test with Bonferroni correction. The illustrated results were representative of three replicates. ****p < 0.0001.

*YTHDF1* also promoted GBC cell migration and invasion in Transwell migration and invasion assays, respectively (Figure 3A). In addition, *YTHDF1* promoted cell migration in wound healing assays (Figure 3B).

Collectively, these results indicated that *YTHDF1* had a critical role in the malignant behaviours of GBC cells.

### 3.3 YTHDF1 promotes gallbladder cancer cell proliferation and metastasis in mice models

**FIGURE 4:**
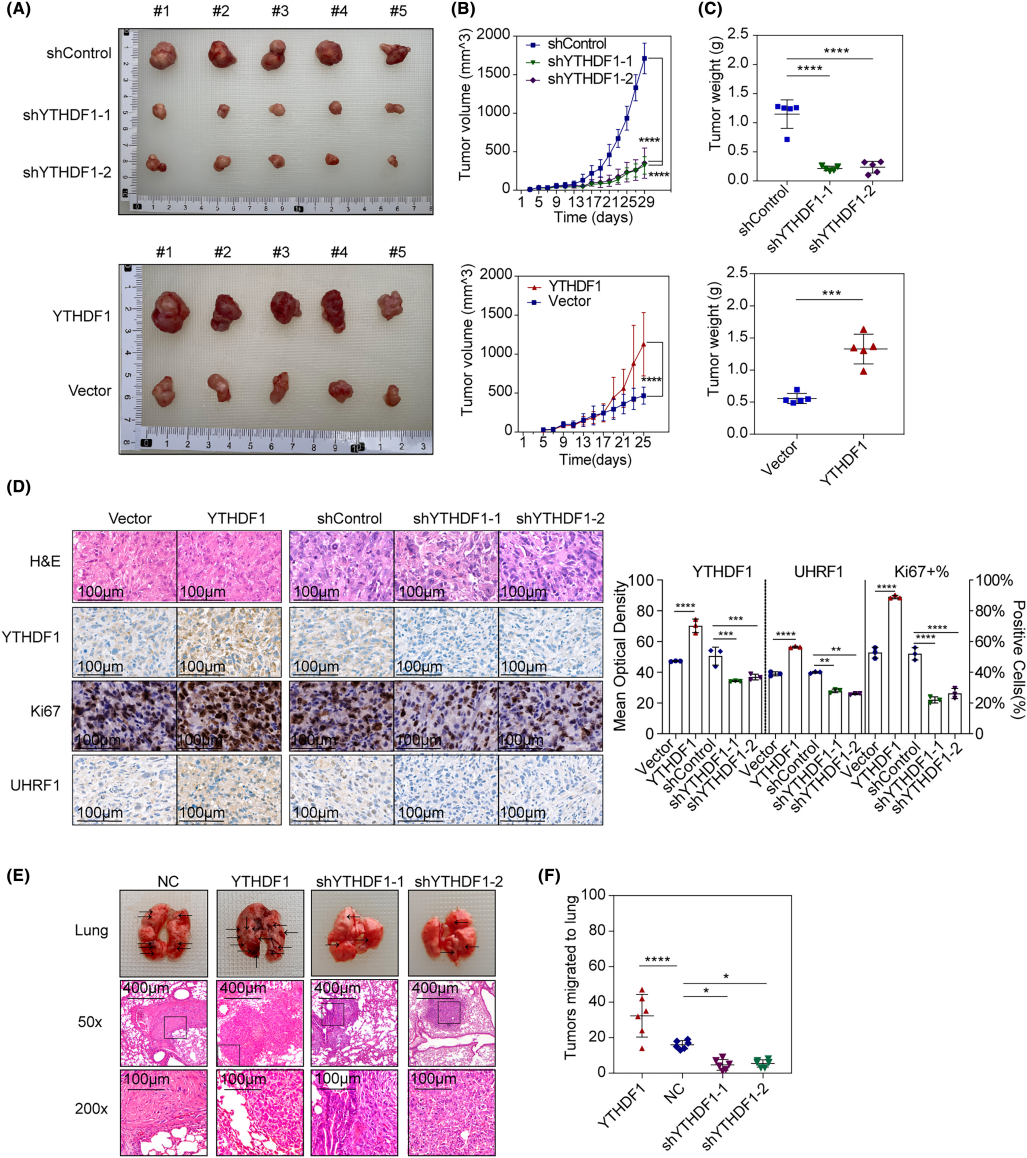
(A) Tumours from the xenograft mice model using YTHFD1‐stably expressing NOZ cells; (B) tumour growth curve of xenograft mice models. Two‐way ANOVA test and Tukey's multiple comparison tests were used to compare between groups. ****p < 0.0001; (C) tumour weight of xenograft mice models. ANOVA test or *t*‐test was used to compare between groups. ***p < 0.001, ****p < 0.0001; (D) H&E and IHC staining of xenograft tumours; the images with 400× final magnification were illustrated; the IHC scores were evaluated using MODs in the DAB channel, and the Ki67 positive cell ratio (Ki67%) was calculated and compared among the groups. Comparisons between groups were made using ANOVA, Tukey's multiple comparison tests, Chi‐square tests with Bonferroni correction. **p < 0.01, ***p < 0.001, ****p < 0.0001; (E) lung tissues of tail vain injection mice model; the images with 50× and 400× final magnification was illustrated; (F) number of tumours migrated to lung in tail vein injection mouse model. ANOVA and Tukey's multiple comparison tests were used to compare between groups. *p < 0.05, ****p < 0.0001.

Administration of cells overexpressing *YTHDF1* increased tumour volumes when compared with Vector group (Figure 4A,B; *p* < 0.0001), while cells knocking‐down YTHDF1 (shYTHDF1‐1, *p* < 0.0001 and shYTHDF1‐2, *p* < 0.0001) decreased tumour volumes when compared with shControl group in the xenograft model (Figure 4A,B). Tumour weights were increased in YTHDF1 overexpression group than in Vector group (*p* = 0.0001), and decreased in shYTHDF1‐1 (*p* < 0.0001) and shYTHDF1‐2 group (*p* < 0.0001) than in shControl group (Figure 4C). These results suggested that YTHDF1 promoted tumour growth in a xenograft mouse model.

We then validated YTHDF1 expression via IHC staining of the harvested tumours. IHC staining of YTHDF1, Ki67 and UHRF1 revealed that the abundance of YTHDF1 and UHRF1 proteins, as well as the percentage of Ki67 positive cells was upregulated in tumour tissues derived from the *YTHDF1*‐overexpressing NOZ cell line and downregulated in those derived from the YTHDF1‐downregulated NOZ cell line (Figure 4D). Hence, YTHDF1 might cause changes in UHRF1 and Ki67 expression in tumour tissues.

In the tail vein injection model, we counted metastatic tumours in the lungs of mice and found an increased number in mice administered *YTHDF1*‐overexpressing NOZ cells (YTHDF1, *p* < 0.001) and decreased number of tumours in those administered *YTHDF1*‐knockdown cells (shYTHDF1‐1, *p* < 0.05 and shYTHDF1‐2, *p* < 0.05) when compared with NC group (Figure 4E,F; Figure S1). These results suggested that YTHDF1 increased GBC growth and metastases.

### 3.4 Multi‐omics analyses reveal that YTHDF1 may function as a regulator of UHRF1

We investigated whether YTHDF1 altered the malignant behaviour of GBC using an *YTHDF1*‐overexpression vector and three distinct short hairpin RNAs (shYTHDF1‐1, shYTHDF1‐2 and shYTHDF1‐3) to alter *YTHDF1* expression in NOZ and GBC‐SD cell lines. RNA‐seq was performed using shYTHDF1‐1–3 and shControl.

**FIGURE 5:**
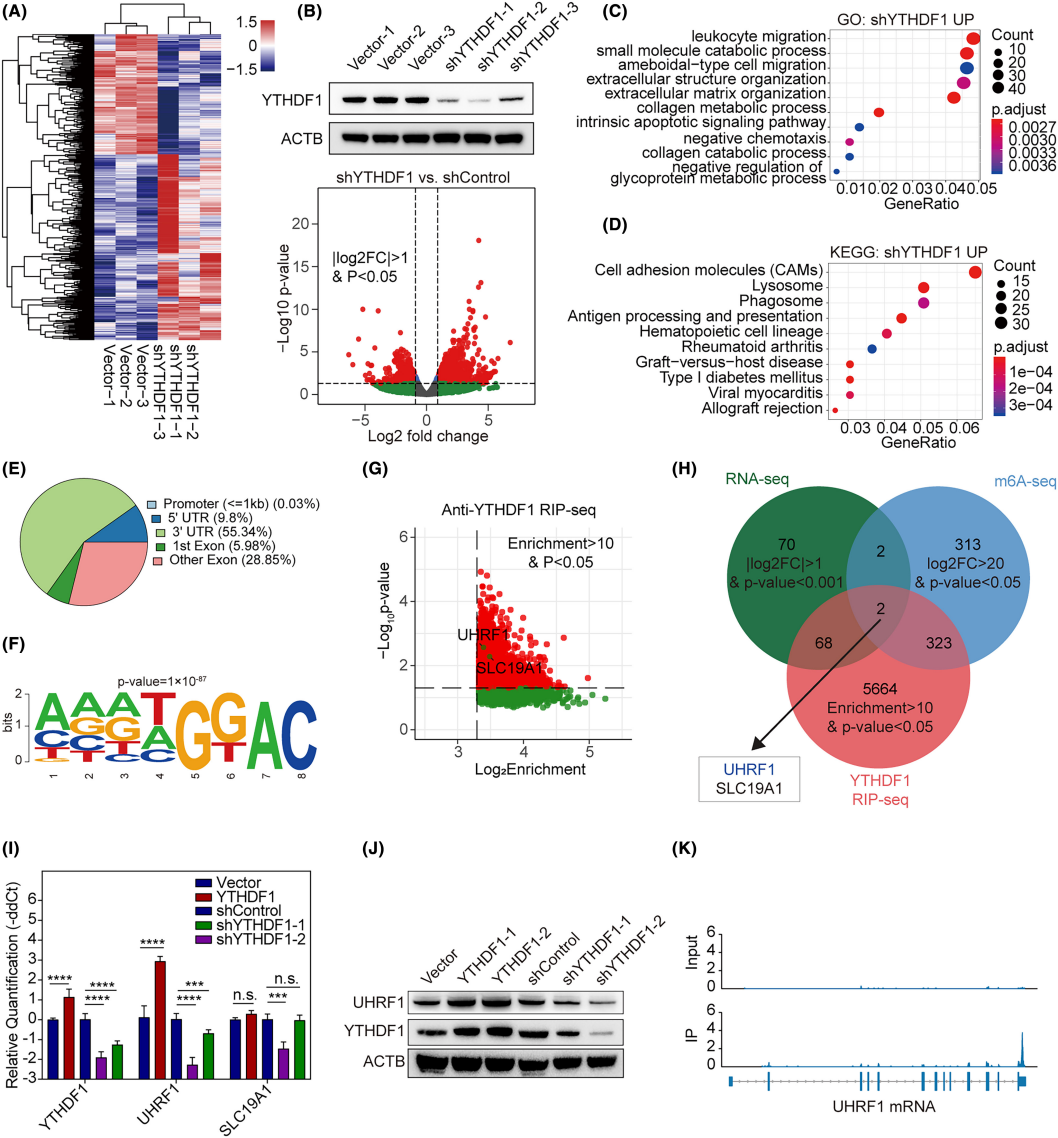
(A) Transcriptome analyses of YTHDF1 knockdown in NOZ cells. Significant genes are illustrated in the heatmap; (B) (up) quality control of YTHDF1 knockdown in NOZ cells; (down) volcano plot of DEGs of *YTHDF1* knockdown. Red dots represent |log2FC|>1 and *p* < 0.05. Green dots represent |log2FC|>1 with *p* > 0.05; (C) gene ontology enrichment analysis of downregulated genes after *YTHDF1* knockdown; (D) KEGG enrichment analyses of downregulated genes after *YTHDF1* knockdown; (E) m6A peak distribution on different regions of mRNA; (F) motif analysis revealing enriched m6A binding motif; (G) plot for enrichment and *p*‐values in anti‐*YTHDF1* RIP‐seq. UHRF1 is represented as blue in the dot plot. Genes with *p* < 0.05 appear red, with *p* > 0.05 appear grey; (H) Venn plot presents significant genes in RNA‐seq, m6A‐seq and RIP‐seq. Overlapping genes included *UHRF1* and *SLC19A1*. (I) Realtime PCR illustrating changes in *UHRF1* and *SLC19A1* mRNA expression after *YTHDF1* alteration; YTHDF1‐1 and YTHDF1‐2 were technical repeats; (J) Western blotting analyses changes in UHRF1 protein abundance after YTHDF1 alteration; (K) m6A peak distribution on UHRF1 mRNA in m6A‐seq. (up) Input sample of m6A‐seq; (down) IP sample of m6A‐seq. Expression level of Input and IP sample are normalized to the same level.

Transcriptomic analysis revealed that *YTDHF1* knockdown altered the levels of 2212 mRNA transcripts, of which 869 were downregulated (Figure 5A,B). Gene ontology analysis further revealed that these positively correlated genes were enriched in multiple biological processes, including the regulation of DNA metabolic processes, DNA replication, ncRNA processing and DNA recombination (Figure 5C). KEGG enrichment analysis showed that YTHDF1 was positively correlated with the cell cycle, Fanconi anaemia pathway and homologous recombination (Figure 5D).

Given that YTHDF1 is an m6A ‘reader’, an m6A‐seq was performed to detect m6A modified targets. Approximately half of the m6A peaks were located in the 3′‐UTR of mRNAs (Figure 5E). Peak motif analysis further identified a 5′‐AAATGGAC‐3′ motif within the m6A‐seq of the NOZ cell line (Figure 5F).

To identify the mRNAs that YTHDF1 directly binds to, we performed RNA immunoprecipitation and sequencing of the NOZ cell line. A total of 6057 genes were significantly enriched in the two replicates of the anti‐YTHDF1 group compared to the 10% input group (Figure 5G).

Multi‐omics analyses then revealed two genes that were significantly affected by *YTHDF1* knockdown, with specific YTHDF1 and m6A binding peaks (Figure 5H), among which, *UHRF1* was found to have a significant m6A peak in the 3′‐UTR (Figure 5K).

The results of real‐time PCR validation demonstrated an increase in UHRF1 expression with YTHDF1 overexpression and a decrease with YTHDF1 knockdown, indicating a potential targeting of UHRF1 by YTHDF1. The expression of SLC19A1 did not exhibit consistent changes when YTHDF1 was overexpressed or knocked down (Figure 5I). Western blotting confirmed that UHRF1 protein levels increased upon YTHDF1 overexpression and decreased upon its knockdown (Figure 5J).

Taken together, *UHRF1* mRNA was a potential target for YTHDF1 and the mechanism may be m6A‐dependent.

### 3.5 The YTHDF1/m6A/UHRF1 axis increases cell proliferation and metastases while inhibits cell apoptosis

**FIGURE 6:**
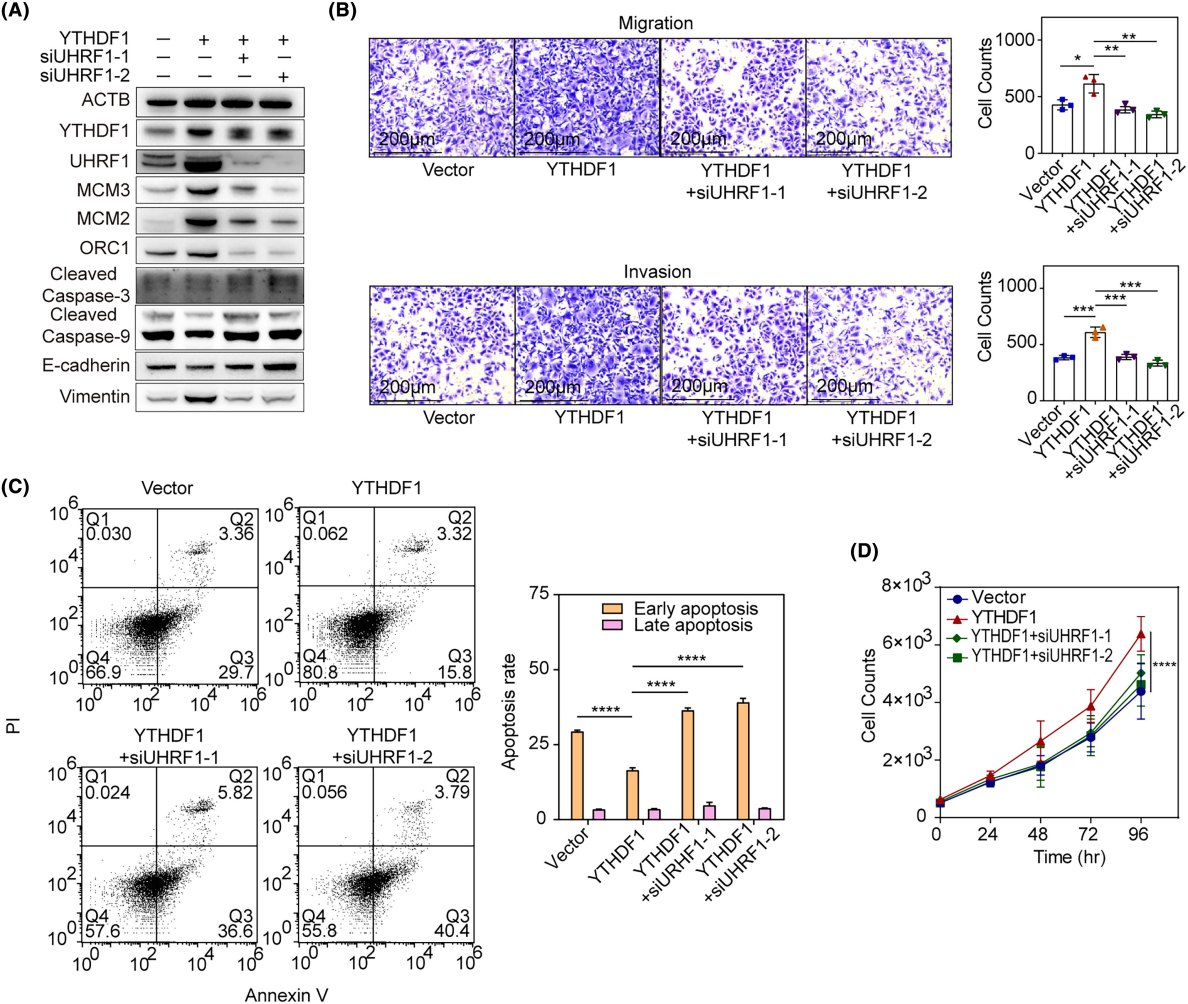
(A) Western blotting of YTHDF1 overexpression and siUHRF1 rescue assays and downstream targeted genes; the illustrated results were representative of three replicates. (B) Rescued cell migration & invasion assay of *UHRF1* knockdown in YTHDF1‐overexpressing NOZ cells; two‐way ANOVA was used to compare multiple curves and Tukey's multiple comparison tests were used to compare groups. The illustrated results were representative of three replicates. *p < 0.05, **p < 0.001, ***p < 0.0001; (C) rescued cell apoptosis assay of *UHRF1* knockdown in *YTHDF1*‐overexpressing NOZ cells; early apoptosis (Q3) and late apoptosis (Q2) was compared between groups using Chi‐square tests with Bonferroni correction. The illustrated results were representative of three replicates. ****p < 0.0001; (D) rescued cell proliferation assay of *UHRF1* knockdown in *YTHDF1*‐overexpressing NOZ cells; two‐way ANOVA test and Tukey's multiple comparison tests were used to compare between groups. The illustrated results were representative of three replicates. ****p < 0.0001.

To establish the functioning of the YTHDF1/UHRF1 axis in NOZ cells, siUHRF1 rescue assays were performed. The findings reveal that siUHRF1 partially reversed YTHDF1‐induced changes in cell proliferation (MCM2, MCM3 and ORC1), apoptosis (cleaved caspase‐3 and cleaved caspase‐9), and epithelial‐mesenchymal transition (E‐cadherin and vimentin; Figure 6A).

In Transwell migration and invasion studies, siUHRF1 partially reversed cancer cell migration and invasion in NOZ cells after YTHDF1 overexpression (Figure 6B). Overexpression of YTHDF1 decreased the rate of CCCP‐induced early apoptosis, whereas siUHRF1 reversed the effects (Figure 6C). YTHDF1 also increased NOZ cell proliferation, while siUHRF1 reversed the changes, respectively (Figure 6D).

### 3.6 YTHDF1 directly binds to the UHRF1 mRNA 3′‐UTR in an m6A‐dependent manner and increases mRNA stability

**FIGURE 7:**
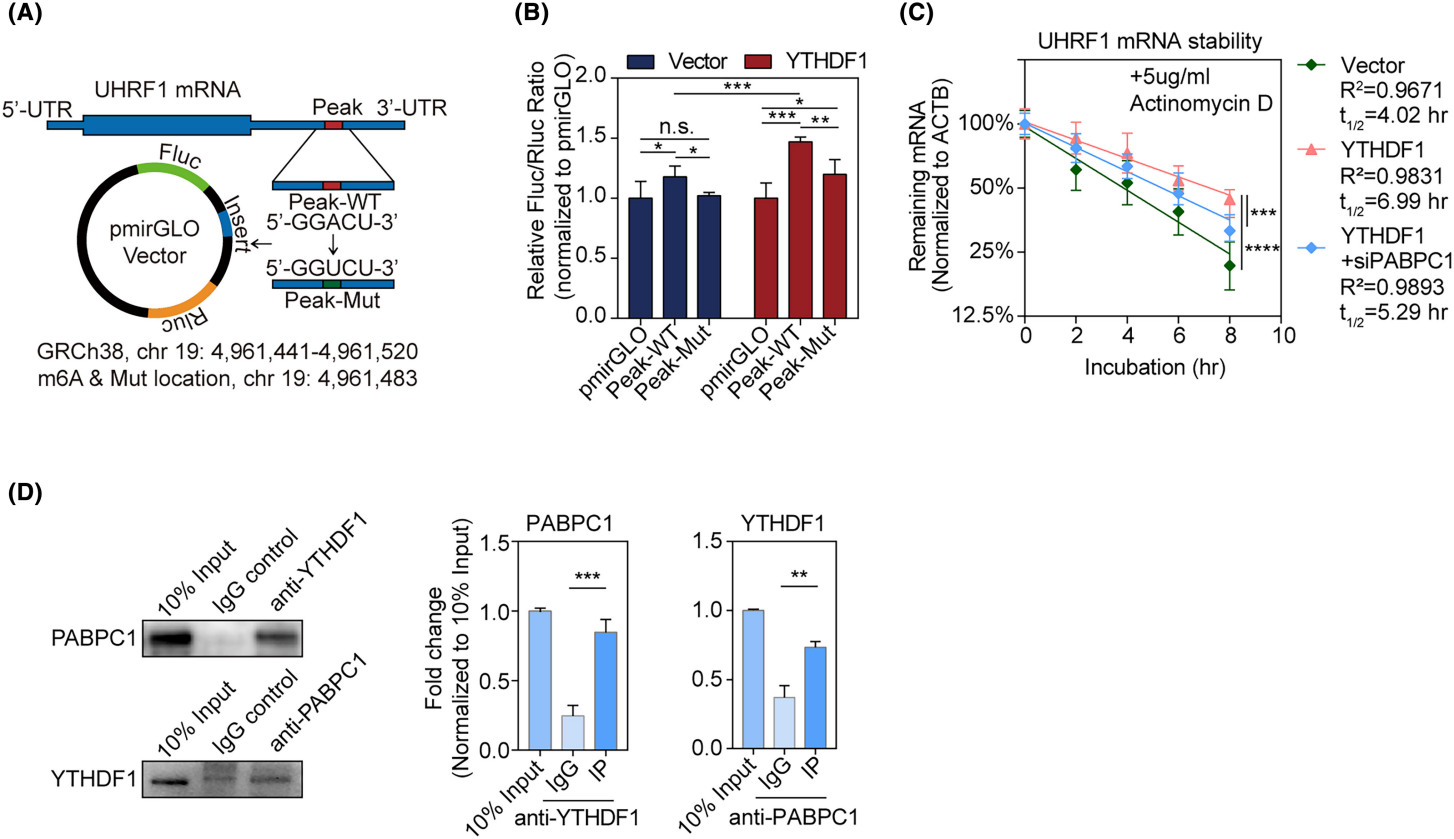
(A) Design of insert sequence in dual‐luciferase assays; (B) dual‐luciferase assay of Peak‐WT and Peak‐Mut in NOZ cells stably expressing vector and YTHDF1. Expression level is normalized to the empty pmirGLO vector. ANOVA and Tukey's multiple comparison tests were used to compare between groups; the illustrated results were representative of three replicates; *p < 0.05, **p < 0.01, ***p < 0.001; (C) *UHRF1* mRNA stability assay of Vector, YTHDF1, and YTHDF1 + siPABPC1 in NOZ cells; A linear regression model was fitted to log2‐transformed mRNA expression and incubation time, and *R*_2_ and *t*_1/2_ were calculated. Comparisons of the different decay rates were performed by two‐way ANOVA; the illustrated results were representative of three replicates; **p < 0.01. (D) Western blotting of YTHDF1 and PABPC1 co‐immunoprecipitation in NOZ cells; ANOVA and Tukey's posthoc tests were used to compare between groups; The illustrated results were representative of three replicates; **p < 0.01.

To determine whether m6A peak in UHRF1 3′‐UTR is a direct target of YTHDF1, dual‐luciferase assay vectors containing a wild‐type m6A site (Seq1‐WT) or mutated m6A site (Seq1‐Mut) in the 3′‐UTR region were established and transfected separately into NOZ cells (Figure 7A). The Fluc/Rluc ratio significantly increased in Seq1‐WT mice. In contrast, the Seq1‐Mut, containing a mutated binding site (GGACU to GGUCU), partially reversed the change in the Fluc/Rluc ratio induced by Seq1‐WT. Compared with the NC cell‐line, *YTHDF1*‐overexpressing NOZ cells manifested a higher Fluc/Rluc ratio in Seq1‐WT and Seq1‐Mut cells. However, Seq1‐Mut partially reversed the Fluc/Rluc ratio change induced by Seq1‐WT in YTHDF1‐overexpressing cells (Figure 7B).

Given that m6A modification may affect mRNA stability, our study utilized actinomycin D‐dependent mRNA stability assays to gain insight into the functions of YTHDF1 and YTHDF1 + siPABPC1 on UHRF1 mRNA. As a result, YTHDF1 overexpression increased the half‐life of UHRF1 mRNA from 4.02 to 6.99 h (Figure 7C), and siPABPC1 reversed the half‐life of UHRF1 mRNA from 6.99 to 5.29 h. YTHDF1‐overexpressed cells had a considerably slower UHRF1 mRNA decay rate (Figure 7C; *p* < 0.0001), and siPABPC1 + YTHDF1 partially reversed the changes (Figure 7C; *p* < 0.001).

Considering PABPC1 has been demonstrated to regulate m6A‐dependent mRNA stability, we used reciprocal co‐IP experiments to determine whether YTHDF1 directly bound to PABPC1 in NOZ cells. As a result, PABPC1 was significantly enriched in anti‐YTHDF1 Co‐IP assays, and vice versa (Figure 7D).

Taken together, these results suggested that YTHDF1 promoted its expression by direct binding to the 5′‐GGACU‐3′ binding site on the 3′‐UTR of *UHRF1* mRNA, and binding to PABPC1 may be significant for YTHDF1 promoting *UHRF1* mRNA stability, and further functioning in GBC.

## 4 DISCUSSION

In this study, we discovered that YTHDF1 increases the expression of *UHRF1* in an m6A‐dependent manner, which ultimately mediates GBC progression. Thus, *YTHDF1* is an oncogene that facilitates GBC cell proliferation, migration, and invasion, while inhibiting cell apoptosis.

GBC is an aggressive cancer that often requires invasive surgery, even at stage T1b.^5^, ^26^ We found that *YTHDF1* expression was significantly higher in grade I/II and grade III GBC tissues than in para‐tumour tissues. Moreover, transcriptome analyses of matched GBC tissues from our cohort and an external dataset revealed YTHDF1 overexpression in the tumour tissues. Our murine models further revealed that administration of cell lines overexpressing *YTHDF1* enhanced tumour growth, elevated Ki67 expression, and increased lung metastasis. Taken together, these findings imply that YTHDF1 is involved in the proliferation and metastasis of GBC.

Multiple functions have been previously described for YTHDF1 in tumour carcinogenesis, including promotion of Wnt/β‐catenin signalling,^27^, ^28^ influencing epithelial‐mesenchymal transition,^8^, ^9^ facilitating tumour immune escape,^29^, ^30^ and increasing chemoresistance and cell cycling.^31^ Meanwhile, in this study, the RNA m6A reader, YTHDF1, enhanced *UHRF1* expression in an m6A‐dependent manner. A multi‐omics analysis, including RNA‐seq, RIP‐seq, and m6A‐seq, further confirmed *UHRF1* as a target gene of YTHDF1 associated with GBC progression, which has not been previously reported.

UHRF1 overexpression epigenetically silenced the tumour suppressor genes in many various solid and haematological tumours.^14^, ^32^ UHRF1 was able to prevent apoptosis in both gallbladder cancer and colorectal cancer cells,^14^, ^15^ promote cell cycle in GBC cells,^14^ promotes osteosarcoma metastasis through altered exosome production and AMPK/SEMA3E suppression,^16^ inducing epithelial‐mesenchymal transition by upregulating CXCR4 in cancer cells,^17^ and promoting aerobic glycolysis.^19^ In conclusion, UHRF1 acted as an oncogene in multiple cancers. In our research, we proposed a novel mechanism of YTHDF1 post‐transcriptionally regulated UHRF1 level that promotes cell proliferation and cancer metastasis while inhibiting cell apoptosis.

YTHDF1 overexpression increased the Fluc/Rluc ratio when transfected with the Seq1‐WT vector containing the *UHRF1* mRNA 3′‐UTR region flanking the m6A binding motif 5′‐GGACU‐3′. In comparison, the Fluc/Rluc changes were significantly reversed by a Seq1‐Mut vector with a mutation in the 5′‐GGACU‐3′ motif (5′‐GGUCU‐3′) compared to Seq1‐WT. Hence, the function of YTHDF1 was determined to be dependent on the specifically identified m6A motif 5′‐GGACU‐3′. Indeed, the 5′‐RRACH‐3′ sequence is reportedly a common m6A‐enriched motif.^33^, ^34^

Previous studies revealed that regulating mRNA stability and translation were essential events in m6A‐related post‐transcriptional regulation. It has been reported that IGF2BP1 enhanced mRNA stability by recruiting PABPC1 to the target mRNA.^35^ YTHDF3 was additionally proven to bind PABPC1 in haematopoietic cells and enhance Ccnd1 translation.^36^ PABPC1 was able to stabilize lncRNA‐PAGBC in gallbladder cancer, and activates the AKT/mTOR pathway to promote tumour growth and metastasis.^37^ PABPC1 interacted with BDNF‐AS and increased its expression by stabilizing the expression of BDNF‐AS, and overexpression of both inhibited proliferation, migration and invasion of glioblastoma and promoted apoptosis.^38^ In our investigation, we discovered that YTHDF1 may also bind to PABPC1 in GBC cells. We propose a novel YTHDF1 mechanism that raises target gene expression.

## AUTHOR CONTRIBUTIONS

**Jiemin Chen:** Conceptualization (equal); data curation (equal); formal analysis (equal); investigation (lead); methodology (lead); software (lead); visualization (equal); writing – original draft (lead); writing – review and editing (lead). **Xuesong Bai:** Data curation (equal); investigation (equal); methodology (equal); visualization (equal); writing – review and editing (equal). **Wenqin Zhang:** Formal analysis (equal); investigation (equal); methodology (equal); visualization (equal); writing – review and editing (equal). **Zhiyu Yan:** Investigation (supporting); software (supporting); validation (lead). **Yongru Liu:** Investigation (supporting); validation (equal). **Shengnan Zhou:** Data curation (equal); validation (supporting). **Xi Wu:** Funding acquisition (equal); project administration (supporting). **Xiaodong He:** Conceptualization (equal); funding acquisition (equal); project administration (equal); resources (equal). **Aiming Yang:** Conceptualization (equal); funding acquisition (lead); project administration (lead); resources (lead); writing – review and editing (supporting).

## FUNDING INFORMATION

This article is under the support of the National Key R&D Program of China (Grant Number 2022YFC3602103) to Y.A.M.; National High‐Level Hospital Clinical Research Funding (Grant Number 2022‐PUMCH‐B‐024) to Y.A.M.; National High‐Level Hospital Clinical Research Funding (Grant Number 2022‐PUMCH‐C‐063) to W.X.; CAMS Initiative for Innovative Medicine (Grant Number 2021‐I2M‐1‐013) to Y.A.M.; CAMS Initiative for Innovative Medicine (Grant Number 2021‐I2M‐1‐022) to H.X.D.; National Key Clinical Specialist Construction Project (Grant Number ZK108000).

## CONFLICT OF INTEREST STATEMENT

The authors confirm that there are no conflicts of interest.

## Supporting information


Data S1.

## Data Availability

The raw sequence data reported in this study were deposited in the Genome Sequence Archive^39^ of the National Genomics Data Center,^40^ China National Center for Bioinformation/Beijing Institute of Genomics, Chinese Academy of Sciences (GSA‐Human: HRA003729, HRA003818), and are publicly accessible at https://ngdc.cncb.ac.cn/gsa‐human. All other research data in this study was available upon reasonable request.
