# Supplementary material for: YTHDF1 promotes gallbladder cancer progression via post‐transcriptional regulation of the m6A/UHRF1 axis
Source: J Cell Mol Med. 2024 Apr 29;28(9):e18328. doi: 10.1111/jcmm.18328 (PMC11057417; doi:10.1111/jcmm.18328)
Supplement: Supplementary file 1 — Data S1. [file JCMM-28-e18328-s001.docx]

*Table S1. Baseline data of enrolled patients.*

|  | YTHDF1-high (n=64) | YTHDF1-low (n=65) | P-value |
| --- | --- | --- | --- |
| Age^1^ | 65.2±9.66 | 65.5±10.28 | 0.865 |
| Gender (Male) | 21 (32.8%) | 22 (33.8%) | 0.901 |
| TNM stage |  |  | 0.918 |
| —Stage I | 3 | 2 |  |
| —Stage IIA/B | 14 | 16 |  |
| —Stage IIIA/B | 27 | 29 |  |
| —Stage IVA/B | 20 | 18 |  |
| Pathologic grade |  |  | 0.605 |
| —Grade I | 3 | 2 |  |
| —Grade II | 37 | 43 |  |
| —Grade III | 24 | 20 |  |
| Liver metastasis | 20 (31.25%) | 23 (35.38%) | 0.618 |
| Lymphovascular invasion | 13 (20.31%) | 17 (26.15%) | 0.432 |

^1^.The age comparison between the two groups was conducted using a Student's t-test. Additionally, the TNM stage, pathologic grade, presence of liver metastasis, and lymphovascular invasion were compared between the two groups using Chi-square tests and Fisher's exact tests.

*Tables S2. Oligonucleotides Used in this Study*

| Name | Sequence |
| --- | --- |
| shYTHDF1-1-F | GATCCGCACTGACTGGTGTCCTTTCTCGAAAGAAAGGACACCAGTCAGTGCTTTTTG |
| shYTHDF1-1-R | AATTCAAAAAGCACTGACTGGTGTCCTTTCTTTCGAGAAAGGACACCAGTCAGTGCG |
| shYTHDF1-2-F | GATCCGGAGAATAACGACAACAAACCCGAAGGTTTGTTGTCGTTATTCTCCTTTTTG |
| shYTHDF1-2-R | AATTCAAAAAGGAGAATAACGACAACAAACCTTCGGGTTTGTTGTCGTTATTCTCCG |
| shYTHDF1-3-F | GATCCGCTCAACCGCAGTATCAGACGAATCTGATACTGCGGTTGAGCTTTTTG |
| shYTHDF1-3-R | AATTCAAAAAGCTCAACCGCAGTATCAGATTCGTCTGATACTGCGGTTGAGCG |
| siUHRF1-1 | GCCAGAGUGAGUCAGACAAdTdT |
| siUHRF1-2 | GCUGGCUCUCAACUGCUUUdTdT |
| pmirGLO-Seq1-WT | GAGAGCAGGGAGTTGGGGCCACGCAGAAATGGCCTCAAGGGGACTCTGCTCCACGTGGGGCCAGGCGTGTGACTGACGCT |
| pmirGLO-Seq1-Mut | GAGAGCAGGGAGTTGGGGCCACGCAGAAATGGCCTCAAGGGGTCTCTGCTCCACGTGGGGCCAGGCGTGTGACTGACGCT |
| siPABPC1 | AUCCAUUGAUAAUAAAGCAdTdT |

*Table S3. Realtime PCR Primers Used in this Study*

| Gene | Forward | Reverse |
| --- | --- | --- |
| ACTB | GGCACCCAGCACAATGAAG | CCGATCCACACGGAGTACTTG |
| YTHDF1 | ACCTGTCCAGCTATTACCCG | TGGTGAGGTATGGAATCGGAG |
| UHRF1 | AGGTGGTCATGCTCAACTACA | CACGTTGGCGTAGAGTTCCC |
| SLC19A1 | AGCTTCATCACCCCCTACCT | TGATCTCGTTCGTGACCTGC |

*Table S4. Antibody Used in this Study*

| Gene / Marker | Antibody | Species | Catalog Number | Manufacturer | Concentration |
| --- | --- | --- | --- | --- | --- |
| ACTB | HRP-conjugated Beta Actin Monoclonal antibody | Mouse | HRP-66009 | Proteintech | 1:10000 |
| ACTB | CoraLite® Plus 488-conjugated Beta Actin Monoclonal antibody | Mouse | CL488-66009 | Proteintech | 1:10,000 |
| YTHDF1 | YTHDF1 Polyclonal antibody | Rabbit | 17479-1-AP | Proteintech | 1:2,000(WB)  1:50(IP)  1:200(IHC)/pH 9 |
| UHRF1 | UHRF1 Polyclonal antibody | Rabbit | 21402-1-AP | Proteintech | 1:1,000  1:50(CUT&Tag)  1:200(IHC)/pH 6 |
| PABPC1 | PABPC1 Polyclonal antibody | Rabbit | 10970-1-AP | Proteintech | 1:1000(WB)  1:50(IP) |
| ORC1 | ORC1 Rabbit pAb | Rabbit | A14756 | Abclonal | 1:1000 |
| MCM2 | MCM2 Polyclonal antibody | Rabbit | 10513-1-AP | Proteintech | 1:1000 |
| MCM3 | MCM3 Polyclonal antibody | Rabbit | 15597-1-AP | Proteintech | 1:1000 |
| MCM4 | MCM4 Polyclonal antibody | Rabbit | 13043-1-AP | Proteintech | 1:1000 |
| Cleaved Caspase-3 | Caspase 3/p17/p19 Polyclonal antibody | Rabbit | 19677-1-AP | Proteintech | 1:500 |
| Cleaved Caspase-9 | Caspase 9/p35/p10 Polyclonal antibody | Rabbit | 10380-1-AP | Proteintech | 1:1000 |
| E-cadherin | E-cadherin Polyclonal antibody | Rabbit | 20874-1-AP | Proteintech | 1:10000 |
| Vimentin | Vimentin Polyclonal antibody | Rabbit | 10366-1-AP | Proteintech | 1:10000 |
| Rabbit IgG Isotype Control | Rabbit (DA1E) mAb IgG XP® Isotype Control #3900 | Rabbit | 3900S | Cell Signaling Technology | 1:100 |
| m6A | Anti-N6-methyladenosine (m6A) Antibody | Rabbit | ab151230 | Abcam | 1:100 |
| Ki67 | KI67 Polyclonal antibody | Rabbit | 27309-1-AP | Proteintech | 1:12000(IHC)/pH 9 |
| Rabbit IgG | CyDye™ NIR-labelled secondary antibody 800 nm | Goat | 29360791 | Cytiva | 1:150,000 |
| Rabbit IgG | CyDye™ NIR-labelled secondary antibody 700 nm | Goat | 29360787 | Cytiva | 1:150,000 |


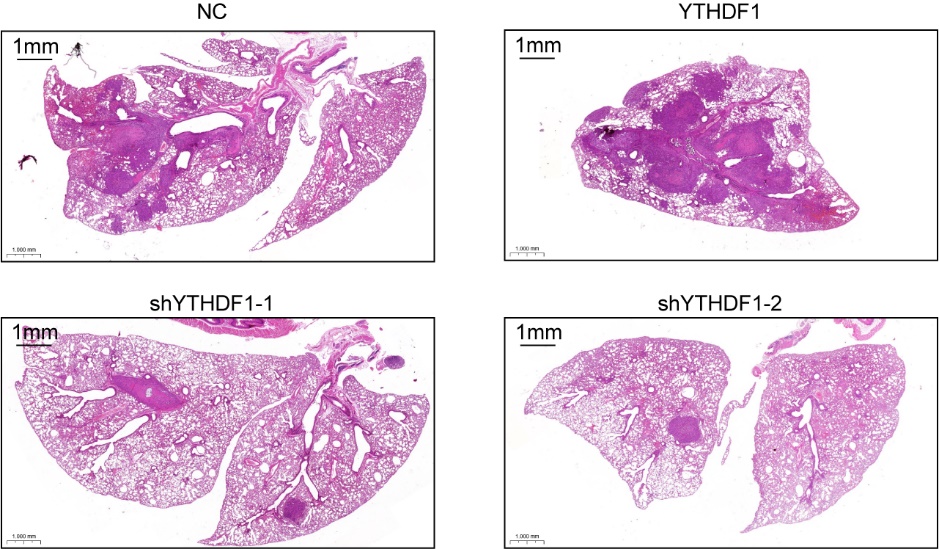


Figure S1. H&E staining of largest section of lung from xenograft mice models. 20x pictures were illustrated and magnification bar was illustrated in captured pictures.
